# Role cognition of assigned nurses supporting Hubei Province in the fight against COVID-19 in China: a hermeneutic phenomenological study

**DOI:** 10.3389/fpsyg.2024.1287944

**Published:** 2024-02-29

**Authors:** Xu Zhang, Yaqian Wang, Yuanyuan Chen, Hailing Yang, Xiaorong Luan

**Affiliations:** ^1^School of Nursing and Rehabilitation, Cheeloo College of Medicine, Shandong University, Jinan, Shandong, China; ^2^Qilu Hospital of Shandong University, Jinan, Shandong, China; ^3^Infection Management Department, Qilu Hospital of Shandong University, Jinan, Shandong, China

**Keywords:** role cognition, coronavirus disease 2019, front-line nurses, nursing experience, hermeneutic phenomenological study

## Abstract

**Aims:**

During the COVID-19 epidemic, nurses played a crucial role in clinical treatment. As a special group, front-line nurses, especially those assigned to support Hubei Province in the fight against COVID-19 between February and April 2020, brought diverse experiences from different provinces in China in taking care of COVID-19 patients and role cognition. Therefore, our purpose is to explore the real coping experience and role cognition of front-line nurses during the novel coronavirus outbreak to provide relevant experience references for society and managers in the face of such major public health emergencies in the future.

**Design:**

This qualitative study was performed using the phenomenological hermeneutics method.

**Method:**

This is a qualitative phenomenological study. Semi-structured in-depth interviews were used to collect data. The interviewees were 53 front-line nurses who assisted and supported the fight against COVID-19 in Hubei Province during the COVID-19 epidemic. Data were collected through individual online and telephone interviews using a semi-structured interview during March 2020. The COREQ guidance was used to report this study.

**Results:**

The findings revealed that front-line nurses assisting in the fight against COVID-19 developed a context-specific role cognition of their work and contribution to society. The qualitative analysis of the data revealed 15 sub-categories and 5 main categories. These five themes represented the different roles identified by nurses. The roles included expectations, conflicts, adaptation, emotions, and flow of blessing. Belief in getting better, a sense of honor, and training could help them to reduce feelings of conflict in this role and adapt more quickly.

**Discussion:**

This article discusses the real coping experience and role cognition of front-line nurses during the novel coronavirus epidemic. It provides relevant experience references for society and managers to face similar major public health emergencies in the future. This study makes a significant contribution to the literature because it demonstrates how non-local nurses sent to Hubei to work perceived their roles as part of a larger narrative of patriotism, duty, solidarity, and hope.

## Highlights

- This study described the experience of nurses from different provinces of China in supporting Hubei Province in combating COVID-19.
- We found that front-line nurses assisting in the fight against COVID-19 developed a context-specific role cognition of their work and contribution to society.
- Finding ways to improve the front-line work environment, help nurses adapt to their new roles, and provide relevant experience for the community and managers facing similar major public health emergencies in the future.

## 1 Introduction

The COVID-19 pandemic has posed an unprecedented threat and challenge to public health worldwide (Zhan et al., 2020). This pandemic broke out in early 2020 and rapidly spread to many countries around the world. The virus was constantly mutating, challenging the capacity of all countries to prevent and control the epidemic. With the joint efforts of governments and people around the world, the epidemic will be well-controlled by 2024. The outbreak of COVID-19 has posed a major challenge to the global healthcare system and has had a significant impact on people’s psychology and bodies.

The Chinese State Health Commission brought it into category B management of legal infectious diseases and took measures to prevent and control Class A infectious diseases. To curb the epidemic, a level I response to major public health events was launched in many places across China. Government officials around the world have accordingly implemented harsh, even extreme measures to combat the spread of the disease (Gupta et al., 2019; Materassi, 2019). It was found that no one had a clear understanding of the new virus (Kalateh Sadati et al., 2020), and all the staff were faced with a mysterious world created by the coronavirus.

However, China did not waver in the face of this unknown virus and the severe test brought to Hubei compatriots. The Chinese government has mobilized massive medical resources, setting up fever clinics, special isolation wards, intensive care hospitals, and temporary mobile cabin hospitals to accommodate all types of infected and asymptomatic patients (Zhou et al., 2024). Hospitals in all provinces and levels responded to the documents and requirements issued by the National Health Commission and selected qualified medical workers in Hubei to support the fight against COVID-19. Many provinces have extended a helping hand to Hubei, and many medical workers have joined the first-line medical teams in the fight against COVID-19, making outstanding contributions to the significant progress in the prevention and control of the epidemic. Thousands of healthcare workers have been involved in the care of patients with confirmed diseases. More than 42,000 medical staff were sent to Wuhan and Hubei, of which 68% were nurses (Zhang, 2020).

Front-line nurses across the country, especially those supporting the fight against COVID-19 in Hubei Province, have carried out heavy treatment and nursing work during the epidemic prevention and control. They were required to work long shifts with inadequate personal protective equipment and constantly learned how to care for patients with viral infections (El-Hage et al., 2020). Under the complex background of high work pressure, high infection rate, and high risk of death, these front-line medical workers perceived uncertainty and fear during the nursing process. Especially in the COVID-19-infected inpatient area, they could clearly observe the unexpected evolution of the disease and the sense of vulnerability in life, which caused their physical and mental health to suffer. In addition, nurses faced the pain of separation from their families, stigma, fear of infection, fear of infecting loved ones, and loss of patients and colleagues (Walton et al., 2020). These extreme conditions could lead to depression, anxiety, insomnia, fear, and somatization symptoms in nurses (Cai et al., 2020).

Studies have shown (Dragioti et al., 2022) that front-line staff working in hospital centers experienced mental health problems such as depressive symptoms, emotional fatigue, and anxiety during the COVID-19 pandemic. Fear-related symptoms, decreased wellbeing, poor quality of life, and acute stress symptoms had the highest prevalence among hospital staff. Nurses were more likely than doctors to experience sleep problems and symptoms of anxiety and depression, with acute stress and insomnia affecting more than half of front-line medical staff. The COVID-19 pandemic has had a significant negative impact on the nursing workforce (Chan et al., 2021). High-intensity and high-pressure work made nurses experience a high level of job burnout during the epidemic of severe special infectious pneumonia (Galanis et al., 2021).

Studies have shown that work-related factors are positively associated with the development of fatigue, job burnout, increased psychological distress, and decreased mental health among nurses caring for COVID-19 patients (World Health Organization, 2020). When the expectations and roles of nurses are inconsistent, role conflicts will easily occur (Tong et al., 2021), which will further affect the efficiency of the team.

Role cognition refers to one’s own understanding of role play and correct cognition of role relationships (Liang et al., 2021). Improving the level of role cognition is conducive to realizing one’s own value and promoting the quality of nursing. Whether an individual can successfully play various roles depends on the degree of cognition of their roles (Xiao et al., 2018). In addition, the number and demand of emergency and critical patients and the diversity and complexity of front-line anti-epidemic nursing work also put forward more new challenges and higher requirements for various abilities of front-line nurses. Therefore, it is necessary to correctly understand the role cognition of front-line nurses in order to play various roles successfully in the complex front-line epidemic nursing work. It has been reported that nurses generally have some problems with role cognition, such as maladaptation, role ambiguity, and role conflict (Wang, 2017). We do not know whether problems in nurses’ role perception are more severe on the front lines where physical and psychological stress is more severe.

No hermeneutic phenomenological study has been conducted on designated front-line nurses who support the fight against COVID-19 in Hubei Province. Role recognition requires deeper exploration and understanding. Therefore, the evaluation of the role perception experience of deployed nurses tends to adopt a qualitative method of hermeneutic phenomenological study. Therefore, the research question guiding this study is “In the early stage of COVID-19 outbreak, what is the role perception, emotional experience, and coping feelings of front-line nurses fighting against COVID-19, and how do they adapt to this role?” [*SIC*] By adopting a phenomenological approach, we aim to explore in depth their experiential and perceived roles as a special group and make valuable contributions to the existing literature.

## 2 Methods

### 2.1 Design

This study used the phenomenological hermeneutics method to deeply explore the real coping experience and role recognition process of front-line nurses fighting against COVID-19 in Hubei Province (Tarrant and Sabo, 2010). Phenomenological hermeneutics was chosen because it can be used to test the basic meaning of experience-based phenomena when interpreting data. Phenomenology reveals the meaning of subjective experiences, while hermeneutics emphasizes the necessary conditions for text interpretation (Gratton and Erickson, 2007). In essence, role cognition needs more in-depth exploration and understanding. Therefore, evaluating non-local nurses’ role cognition experience is more inclined to adopt the qualitative method of phenomenological hermeneutics. This study adopted COREQ comprehensive standards for research reporting to ensure transparency and rigor of research results, such as research team and reflexivity, study design, and data analysis, which were reported in accordance with the COREQ research reporting omnibus standards, as detailed in Supplementary Table S1.

### 2.2 Theoretical framework

Role theory is defined as a social theory that uses roles to explain individual behavior. In the 1920s, American psychologist Mead introduced the concept of roles into social psychology theory (Jin, 2005). Role theory is a social psychology theory that explains the generation, development, and change of social psychology and behavior from the attributes of human social roles. The theory holds that people’s psychosocial and social behaviors are inseparable from their social roles and that people have a recognized expected role for certain roles. Role theory has been widely used in many fields, such as solving behavioral problems in vocational training, strengthening professional ethics, improving family and social interpersonal relations and school education.

In role theory, the individual’s role is the core explanation of human behavior (Rizzo, 1970). Role cognition refers to individuals continuously processing social role norms and evaluating information related to their identity and unique sociocultural attributes. In the process of psychologically determining the corresponding social response model (that is, the rights, obligations, and behavior patterns of specific roles), role cognition plays a key role in individuals playing social roles. It determines the results of individual role behavior practice (Miles, 1977). Role cognition includes role expectations, adaptation, conflicts, and emotions. Role clarity can be achieved by defining individual roles (Willard and Luker, 2010).

Nurses often have a variety of roles, such as caregivers, educators, mentors, and researchers. Due to the shortage of front-line medical staff, front-line nurses needed to work for long hours, and their workload was much more than their usual work. Long working hours, shift work, and increased workload were related to the physical and psychological pain of nurses (Liu et al., 2020). Studies have shown that when nurses have role conflicts, their happiness decreases, work pressure increases, negative emotions for work increase, and turnover intention increases (Kachie et al., 2023). Good role cognition helps to improve the negative emotions, role stress, and subjective experience of nurses (Chen and Changying, 2019). Improving the level of role cognition is conducive to realizing their own value and promoting the quality of nursing.

### 2.3 Setting and sample

This study adopted the phenomenological research method of qualitative research. Semi-structured interviews combining face-to-face and telephone interviews were used to collect data.

The research population in this study was the front-line nurses fighting against the epidemic in Hubei Province. By purposive sampling method and maximum difference sampling strategy (Patton, 2002), front-line nurses from six public hospitals in Shandong Province were selected. The differences were reflected in education level, age, gender, marriage, working years, and other aspects, such as different education levels (doctor’s degree, master’s degree, and bachelor’s degree) and 4–29 years of experience. The sample size was set to interview two more respondents after their data appeared repeatedly, and no new themes were presented as standards during data analysis.

To implement purposeful sampling and identify suitable participants, researchers have actively worked with relevant hospital care departments to contact potential participants by telephone. The researchers explained the purpose and process of the study and ensured the confidentiality of the data. Individual interviews were arranged with the consent of the participants. All 53 patients agreed to participate in the study, provided written informed consent, and were informed of their right to withdraw unconditionally at any time. To protect the privacy of the participants and the confidentiality of the data, the code name was used instead of their real name in the full text.

The inclusion criteria were as follows: (1) nurses who left their hometown to assist in Hubei Province; (2) engaged in clinical care in the Hubei isolation ward; (3) voluntary participation in this research; and (4) able to communicate in Chinese. Exclusion criteria were as follows: nurses involved in other similar studies. Participants were between 26 and 48 years of age; the mean participant age was 35.1 ± 5.6 years. Of all the participants, 43 were women and 10 were men, and all were university graduates. The average working experience was 11.9 ± 6.2 years, ranging from 4 to 29 years. Demographic data are shown in Table 1.

**Table 1:** Demographic characteristics of the participants (*N* = 53).

Characteristics	*n* (%)
Age (years)	
Mean ± SD	35.1 ± 5.6
55–40	14 (26.4)
39–30	29 (54.7)
29–20	10 (18.9)
Sex	
Male	10 (18.9)
Female	43 (81.1)
Family status	
Single	3 (5.7)
Married	49 (92.5)
Divorced	1 (1.9)
Position	
Clinic nurse	43 (81.1)
Manager	6 (11.3)
Other	4 (7.5)
Education	
Bachelor’s degree	39 (73.6)
Master’s degree	7 (26.4)
Working years	
Mean ± SD	11.9 ± 6.2
≥20	6 (11.3)
19—10	26 (49.1)
9—5	19 (35.8)
<5	2 (3.8)
Department	
ICU/CCU/RICU	17 (32.1)
Infectious ward	2 (3.8)
Respiratory	6 (11.3)
Medical ward	26 (49.1)
Other	2 (3.8)

### 2.4 Data collection

All data were fully anonymized and collected by personal interview using a semi-structured interview guide, focusing on nurses’ role cognition experiences. The interview outline was finalized based on previous literature review, theoretical analysis, consultation with experts in related fields, and discussion and revision based on pre-interview results. The interview guide included several open-ended questions such as: “Why did you choose to go to Hubei as a backup?,” “What were your expectations of going to Hubei?,” “What kind of person do you see yourself as?”

Details are provided in Supplementary Table S2.

Interviews were conducted from 12 March to 3 June 2020. Data were collected by YQW and XZ researchers, both of whom had been trained in the same qualitative research methods. Before the interview, the interviewers were contacted in advance to determine a reasonable interview time. Researchers have also clarified research values and attitudes to respondents, gaining trust and ensuring that respondents can accurately express their views. The interview questions were open-ended and non-directed, reducing the possibility of introducing interviewer bias. After semi-structured interviews with 51 participants, data saturation, a state in which no new information is available and coding becomes infeasible (Lewis, 2018), was reached, after which two additional participants were interviewed.

During the interview, the order of the interview outline was adjusted flexibly according to the interview outline and the situation of the interviewees. The interviewers listened carefully, captured the key information, and asked appropriate questions without any induction, suggestion, or evaluation. Participants were encouraged to speak freely according to the outline and explore the deep inner feelings of the interviewees. To expand on the answers, a few follow-up questions meant to generate in-depth responses were asked, such as “Can you elaborate?” and “Would you mind explaining that again?” Crying and silence were allowed during the interview. During the interview, recording equipment was used to record the interview process. At the same time, the expressions, emotions, and other non-verbal behaviors of the interviewees were observed during the face-to-face interview to supplement the transcripts.

Face-to-face interviews were conducted in a meeting room with good privacy to ensure a quiet, spacious, and undisturbed environment, with only interviewers and interviewees present. The telephone interview was conducted in a quiet room with a good Internet communication signal. Interviews were recorded and varied in duration, lasting 30 to 40 min, with an average duration of approximately 35 min.

### 2.5 Data analysis

After each interview, the recordings were transcribed verbatim into transcripts within 24 h. Accuracy was also checked by repeat listening within 24 h of the interview. Data analysis and data collection were performed simultaneously. The study used NVivo 12.0 software for importing, organizing, and exploring data for analysis. NVivo is a Computer-Assisted Qualitative Data Analysis Software. Code names, code definitions, and exemplary quotes were constructed in the code book using the data analysis management tool NVivo 12.0.

The text was then coded and analyzed according to Colaizzi’s method (Colaizzi, 1978). Colaizzi provides a systematic and clear approach, allowing researchers to set aside their views on the phenomenon and understand the experiences of participants. The method also provides opportunities for participants to confirm their statements and is a qualitative method to ensure the credibility and reliability of the results (Sugden and King, 2021).

The challenge of using this method is that the researchers must be impartial in the process of interviewing and analyzing to avoid understanding the phenomenon based on their own personal values and personal tendencies. In addition, because this study was conducted in the early stage of the epidemic, the whole society was in an atmosphere of anxiety and tension. Therefore, it is particularly important and difficult to avoid the potential influence of researchers’ preconceived ideas on the results. During the interview and the analysis for this study, the researcher was fully aware of personal experiences and often reflected on the potential impact that individual factors might have on the results to ensure maximum quality control.

XZ, YQW, HLY, and YYC jointly participated in data analysis and coding as follows: First of all, they read the full transcript of each participant’s interview multiple times to gain a comprehensive understanding of the experiences and perspectives of the familiar participants. In the second step, they analyzed the data word-by-word to identify and extract statements that were significant to the research question. These statements were often in the form of direct quotes from participants. In the third step, they identified and stated the meaning of the substantive statement. In the fourth step, they then searched for common concepts and organized the formulated meanings into categories and clusters of themes. In the fifth step, they exhaustively described the phenomena under investigation by adding typical original statements from participants. In the sixth step, they put similar themes and their descriptions together for repeated comparisons to arrive at similar ideas and construct themes, capturing the essence of participants’ experiences or perspectives. Finally, the resulting theme structure was presented to the participants for examination, and if there were any biases, the researcher would have to step back to re-analyze the data from the first step. In the process of data analysis and coding, when the four coders had different opinions, the research group met to discuss and determine the final result. During this process, the research group had met for a total of two times.

### 2.6 Quality control

There are four quality control criteria in the study: credibility, transferability, dependability, and confirmability (Lincoln and Guba, 1985). The rigor of this study was ensured in the following ways: In the analysis of the data after the interviews, the description of the participants was considered, and it was ensured that the opinions of all participants were reflected in the Results section. Preliminary results were shared with the participants, and their feedback was solicited to validate the findings to ensure the credibility of the study. Transferability was ensured by providing adequate contextual information (setting, sample, sample size, sample strategy, sociodemographic, inclusion and exclusion criteria, and interview procedures). Based on the previous literature review and theoretical analysis, the interview outline was finally determined by consulting experts in related fields and multiple discussions and revisions according to the results of the pre-interview. Interview subjects were selected to assess saturation in strict accordance with inclusion and exclusion criteria to ensure that a sufficient amount of data was collected to capture the breadth and depth of the research topic. Four researchers checked raw and transcribed data, independently coded, classified, and extracted themes, jointly completed data analysis, and before reaching an agreement, a triangulation method was used to discuss any differences and disagreements on themes, ensuring consistency and contributing to the credibility of the qualitative study. Each research member had a master’s degree or above and participated in qualitative research training. In the process of implementation, relevant nursing managers, psychologists, public health personnel, and nurses in Hubei Province were invited to carry out consultation and thematic communication to obtain valuable insights and improve the credibility of the study. During the member inspection, we should understand the feelings and experiences of each person, understand the research implementation process, and promote the self-reflection of researchers. We should be aware of their biases and reflect on their influence during the whole research process, correct subjective bias in time, reduce research bias to solve reflexive problems, and improve the reliability, credibility, and certainty of research as much as possible.

### 2.7 Ethical considerations

The study was conducted in accordance with the Declaration of Helsinki and was approved by the Committee of Qilu Hospital, Shandong University (approval number: KYLL-2020-258). Before the interview, participants were informed about the purpose of the study, after which they were asked to provide verbal or written consent. Participants were also informed about the privacy of their contributions and their right to withdraw from the study at any time. At the same time, we code-transformed the names and hidden information of the respondents.

## 3 Results

Participants were aged between 26 and 48 years; 43 were women and 10 were men. The participants had a mean age of 35 years and a mean of 11.9 ± 6.2 years of working experience. Their working departments included the intensive care unit (ICU)/cardiovascular intensive care unit (CICU)/Respiratory Intensive Care Unit (RICU), infectious ward, respiratory, medical ward, and others accounting for 32.1, 3.8, 11.3, 49.1, and 3.8%, respectively. Characteristics of interviewed participants are shown in Table 1. Qualitative analysis of the data revealed 15 sub-themes and 5 main themes, shown in Table 2.

**Table 2:** Overview of themes and sub-themes.

No	Themes	Sub-themes
1	Role Expectations	I want to be the soldier in white
		It is my mission as a party member
		It’s my honor to aid front-line nursing
		We are family
2	Role Conflicts	It’s not easy to work in protective gear
		I’m sorry for my family I neglected
		Stretched thin
3	Role Adaptation	Overcome the inadaptability of protective gears
		A team leader is like the sun in the ICU
		More than just a nurse
4	Role Emotions	You never walk alone
		Hope-The brightest star in the dark
5	Flow of Blessing	Medical staff and patients become the whole one
		Spiritual happiness
		Empathy experience

### 3.1 Role expectations

For 1.4 billion Chinese people, the Spring Festival of 2020 was destined to leave a vivid and profound memory: no family gatherings and dinners, no bustling temples, and no crowded bazaars. During the Spring Festival of 2020, everyone closed their doors and isolated themselves inside, and the streets were empty and full of tension. On the eve of the Chinese New Year’s family reunion, hospitals across China reached out to Hubei, recruiting tens of thousands of nurses to the front lines of the epidemic to help the province fight COVID-19. What encouraged them to go was the responsibility of being a soldier in white clothes, the responsibility of being a Communist Party of China, and the Chinese heart of “when one is in trouble, all sides will support.” At this time, what occupied their hearts was not only the fear of facing the unknown virus but also the good expectation of positive struggle and victory against the epidemic.

#### 3.1.1 Sub-theme 1: I want to be the soldier in white

Nurses were called “angels” and “soldiers in white,” just like “Florence Nightingale.” Nightingale’s spirit of “protecting life, dedication, professionalism, and extraordinary courage” will always exist in the heart of every soldier in white. Most of the respondents thought it was an honor to help Hubei and a nurse’s responsibility to fight against the epidemic. The following are quotes that reflect this theme:

“The epidemic is an order, prevention and control is a responsibility, and marching is a responsibility. Defeating it is our sacred mission. It is my duty to stand up when our country and people need us.” (Nurse35 Age:42 Female Experience:23).

“A hero should be the one who gives the most and gets the least. Our nursing colleagues deserve this title.” (Nurse18 Age:35 Male Experience:5).

“Even I heard of some death cases because of this virus, I was willing to join the team to aid Hubei Province in controlling the epidemic. I thought that was our duties, just like Florence Nightingale. Anxiety come from uncertainty but I believe in our country.” [*SIC*](Nurse3 Age:28 Male Experience:6).

“As a medical worker, when ordinary people are worried and try to stay away, we must deal with this kind of novel coronavirus closely and act as a special ‘retrograde’.” (Nurse32 Age:32 Female Experience:9).

“During the SARS period 17 years ago, we were protected by others, today, we ‘post-90s’ come to protect you.” (Nurse39 Age:40 Female Experience:16).

“Confronted by an urgent epidemic situation, it is not only an unshakable responsibility to help but also an important life experience.” (Nurse40 Age:35 Female Experience:13).

“When SARS hit in 2003, I was on the front line. In January 2015, I went to West Africa to help them treat Ebola patients. Now, I must be there in Hubei!” (Nurse52 Age:38 Female Experience:14).

#### 3.1.2 Sub-theme 2: it is my mission as a party member

“I am a party member, and I remember the oath I took when I became a party member.” More than half of these front-line nurses fighting against the epidemic have the identity of party members. They responded to the call of the Party Central Committee for the first time, stood up, rushed forward, and faced difficulties. Regardless of their own safety, they rushed to the epidemic area with the “most beautiful retrograde” and regarded the fight against the epidemic as a mission to help the people of Hubei out of danger. They said it was their duty to put the needs of the organization first, the interests of the country first, and personal safety second, which is the most basic requirement for a party member. Some statements included the following:

“I did not hesitate to sign up to join the first echelon.” (Nurse26 Age:35 Female Experience:10).

“Our party, which has a history of a hundred years, has never been a community of interests. We come together voluntarily because we share the same beliefs.” (Nurse18 Age:35 Male Experience:5).

#### 3.1.3 Sub-theme 3: it is my honor to aid front-line nursing

Each of the front-line nurses fighting against the epidemic has the heart to fight for the happiness of all the people and the prosperity of the motherland. In the front line of medical treatment, they were fearless and fought with death. They said, “It is our honor to leave the danger to ourselves, to give safety to others, and to protect life with life.” Some statements included the following:

When we asked him what it was like to wear a thick protective suit, his reply was unexpected. He stated, “I think the younger generation should charge ahead and not back down,” he continued, “but it’s just that the N95 mask hurts a little bit.” (Nurse14 Age:31 Female Experience:10).

“I’m willing to protect my country with my life!” (Nurse16 Age:33 Male Experience:4).

“We were here to save lives.” (Nurse20 Age:42 Female Experience:14).

“The day of departure happened to be my 25th birthday, which was the best birthday commemoration of my life.” (Nurse39 Age:40 Female Experience:16).

“When holding Dean Yu’s hand tightly, I felt the spirit of the oncology hospital was passed on to us.” (Nurse41 Age:29 Male Experience:6).

#### 3.1.4 Sub-theme 4: we are family

In the spiritual world of the Chinese people, the country and the family, the collective and the individual are inseparable. Home and country are one, home is a small country, and the country is tens of millions. Where there are the needs of the motherland and the people, there is the true dedication of “giving up a small home for everyone.”

Other statements reflected a family-like sentiment toward the deployment:

“Hubei is like a little boy who is ill, we are brothers or sisters and we are willing to go there to help him.” (Nurse41 Age:29 Male Experience:6).

“All the preparations were for this moment, and we could finally fully play to the strength of our medical staff.” (Nurse45 Age:33 Female Experience:7).

“When SARS happened in 2003, I was a graduate student. They protected us at such a dangerous time. Now, we will protect the home!” (Nurse 49 Age:42 Female Experience:19).

### 3.2 Role conflicts

There was a shortage of front-line medical staff, and front-line nurses fighting against the epidemic were faced with a heavy workload, shortage of protective materials and human resources, changes in patient condition and death, discomfort of heavy protective equipment, separation from family, fear of infection, etc. They were under great pressure and panic, which brought a lot of physical and psychological discomfort and challenges.

#### 3.2.1 Sub-theme 1: it is not easy to work in protective gear

Front-line nurses served in the isolation ward where the virus was diffused. To avoid being infected by the virus, they had to constantly wear protective clothing and equipment. Although the protective equipment ensured their safety, it also restricted the senses of the nurses, such as vision, smell, and touch, greatly altering their perception of the external environment and causing discomfort to the body.

Several nurses discussed the difficulty of working in protective gear. Below are some of their statements:

“On the one hand, I had to get used to the heavy protective clothing that is too stuffy to breathe, while I had to face the slightest carelessness of high-intensity work.” (Nurse10 Age:33 Male Experience:5).

“Although I have worked numerous day and night shifts in the ICU before, I knew that this day shift was different from anything I’ve ever faced before, and I would have close face-to-face contact with the new coronavirus.” (Nurse12 Age:31 Female Experience:11).

“Suddenly nausea obviously worsened, I felt chest tightness and dizziness, my colleagues reminded me to slow down, take a deep breath and adjust slowly.” (Nurse13 Age:43 Female Experience:20).

“On the day I first entered the ward, when I put on my protective suit, N95 mask, and goggles, I immediately felt suffocated, as heavy and slow as wearing a spacesuits.” (Nurse17 Age:29 Female Experience:9).

“I spent nearly five hours in the isolation ward wearing protective clothing, with hazy goggles, blurred vision, and difficulty breathing.” (Nurse18 Age:35 Male Experience:5).

“Yesterday was my second night shift, the feelings ranged from strange to familiar, from worry to rest assured.” (Nurse32 Age:32 Female Experience:9).

“After work every day, I wash my face with alcohol, scrub the nasal cavity and external auditory canal with alcohol cotton swabs, and then wear a single-layer surgical coat in a room temperature of 4°C. The cold feeling of the alcohol bath!” (Nurse2 Age:44 Female Experience:25).

#### 3.2.2 Sub-theme 2: I am sorry for my family I neglected

Most front-line nurses have parents, children, and husbands at home. They are a nurse in society, but they are children, parents, wives, or husbands in the family. At the beginning of the outbreak, they came forward regardless of their own safety and went to the most dangerous front-line support of the epidemic to treat the patients infected with COVID-19. They said that “they tried to fulfill their duties as a nurse, but neglected their responsibilities as a family member.”

Some statements included the following:

“My mother said, ‘The baby is only four months old and still needs you to breast-feed. do you know how worried I will be about you? Have you ever thought that your baby is too young for you to be in danger?’” (Nurse47 Age:37 Female Experience:12).

“Grandpa suddenly passed away, and I did not even have a chance to see him one last time.” (Nurse41 Age:29 Male Experience:6).

“We originally planned to marry and hold a wedding during the Spring Festival, but now we have more important things to do.” (Nurse52 Age:38 Female Experience:14).

“When I received the departure call, it was Lunar New Year’s Eve. I left a table of New Year’s Eve dinner and my family, I felt so sorry for them. But if this outbreak was controlled, it is beneficial for everyone.” (Nurse16 Age:33 Male Experience:4).

“Tears soaked my eyes when I thought of my young son and sick father. However, I made the right choice.” (Nurse17 Age:27 Female Experience:5).

#### 3.2.3 Sub-theme 3: stretched thin

Due to the shortage of front-line medical staff, they had to work longer hours to make up for the staffing gaps, so they faced an overwhelming workload, plus the atmosphere in the isolation unit was grave, the treatment was intense, the patients’ conditions were dangerous and full of uncertainty, the nurses were at high risk of infection, and each nurse was a thin man.

“The patient’s condition changed rapidly, less than a few hours, and the gentlemen who talked with me had already been intubated.” (Nurse15 Age:48 Female Experience:29).

“The workload of nurses was very heavy, with a total of more than 40 patients, only two nurses were responsible, and those two nurses were responsible for the treatment and daily life of more than 40 patients. You can imagine the intensity of the work.” (Nurse11 Age:40 Female Experience:19).

“I was very careful to take off my protective clothing for fear of contaminating myself and affecting my teammates. As long as one person was infected, the whole team was quarantined, and the price was too heavy to bear.” (Nurse18 Age:35 Male Experience:5).

“Everyone was completely silent, maybe everyone was as nervous as I was.” (Nurse12 Age:31 Female Experience:11).

“Although there was no hail of bullets, there was a lot of danger.” (Nurse29 Age:43 Female Experience:23).

### 3.3 Role adaptation

This was despite the many challenges on the job, including staff shortages, continuous long shifts, inadequate safety measures, high stress, and a high risk of death. While trying to overcome these obstacles, the hardships and difficulties they encountered may weaken their resilience and determination at any given time, leading them to consider giving up the fight against the epidemic. However, despite the difficulties, they turned the difficulties into motivation and firmly stuck to the front line of the fight.

#### 3.3.1 Sub-theme 1: overcome the inadaptability of protective gears

Wearing protective equipment to work brought great challenges to nurses: not only physical discomfort but also psychological stress. Most of the respondents said that “with the increase of wearing times and the change of mentality, they gradually adapted to the wearing of such protective equipment, and these protective equipment gradually became their own armor.”

“Teammates said, ‘You with short hair, for infection requirements, are more like a fearless warrior!’” (Nurse48 Age:26 Male Experience:5).

“In the isolation ward, as a result, adult diapers have become one of our protective ‘artifacts’” (Nurse33 Age:26 Female Experience:5).

“I have had many different clothes, of which the most expensive one I have ever worn is the ICU protective clothing. Now, I have used this dress gown.” (Nurse36 Age:38 Female Experience:16).

“I had a headache, vomiting, dizziness, palpitation, and other discomforts a few days ago. Now I learn to breathe slowly and operate slowly, so I feel much better.” (Nurse21 Age:30 Female Experience:11).

“To avoid unnecessary infection, everyone lived in a single room, but we could communicate through We Chat.” (Nurse1 Age:42 Male Experience:10).

“When I saw my colleagues were still struggling to persevere, it strengthened my confidence that persistence was a victory; thousands of patients were still waiting for care.” (Nurse16 Age:33 Male Experience:4).

“Although everyone is sweating and too tired to talk after work every day, everyone is holding on.” (Nurse25 Age:37 Female Experience:19).

“There is only one thought in my mind: hold on!” (Nurse16 Age:33 Male Experience:4).

#### 3.3.2 Sub-theme 2: a team leader is like the sun in the ICU

Managers played an important role in the team, where they had important responsibilities, such as assessing the changing situation of the epidemic, guiding and coordinating different teams, dealing with unexpected situations in the ward, making important treatment decisions, and taking care of every member of the support team.

“As a team leader, I will try my best to ensure the safety of everyone, urge them to complete their tasks, pay attention to their psychological pressure, and help them overcome difficulties.” (Nurse15 Age:48 Female Experience:29).

“I need to familiarize my team members with the workflow and the use of instruments as soon as possible to improve their professional level.” (Nurse27 Age:37 Female Experience:15).

“At first, there was a shortage of materials, so we have to find a way to solve the problem of materials.” (Nurse17 Age:29 Female Experience:9).

“‘I’m a head nurse. I have been working for 29 years, believe me!’ Seeing that the old man did not respond, I firmly grasped the old man’s hand and conveyed faith to him with firm eyes.” (Nurse45 Age:33 Female Experience:7).

#### 3.3.3 Sub-theme 3: more than just a nurse

The nurse in an isolated ward was more than just a nurse. They were also psychologists, social workers, physicists, healthcare assistants, mothers, and so on. They were friends in most cases.

“This reminded me of the Crimean War in this fighting COVID-19, we are the descendants of Nightingale. In the past 200 years, nursing has been developing, care is the essence of nursing, this has never changed and will never change.” (Nurse33 Age:26 Female Experience:5).

“In such difficult times, what we needed to do is to take good care of them, encourage them constantly, and bring them hope to live.” (Nurse5 Age:35 Female Experience:8).

“In the isolation ward, we created various gestures to facilitate communication between doctors, nurses, and patients, even in the local dialect.” (Nurse44 Age:26 Female Experience:4).

“We have developed a nurse–patient communication book, with pictures and words, which are convenient for understanding when the language is not clear.” (Nurse6 Age:33 Female Experience:10).

“We have made the QR code education message, and after scanning the QR code, the patient can see a health education video.” (Nurse7 Age:39 Female Experience:18).

“We are the people that patients can rely on and trust.” (Nurse47 Age:37 Female Experience:12).

### 3.4 Role emotions

The medical workers on the front line were brave and fought against the virus, but they were never alone. They had the solid protection of the country and the full support of the society. Thanks to the efforts of the whole society, new equipment had been put into use to increase production and expansion on the front line of the fight against the epidemic, and batches of protective materials and living materials were sent to Hubei to ensure the basic safety of front-line personnel. Working together and fighting the epidemic together, we will win sooner or later.

#### 3.4.1 Sub-theme 1: you never walk alone

They said that although the work situation was difficult, fortunately, the patients here, our colleagues, family and friends, and the government all gave us full support, and we never walked alone.

“Wearing protective clothing, we could not hug. The most common thing we did was to put up our thumbs or clench our fists to make a victory gesture to encourage.” (Nurse37 Age:27 Female Experience:5).

“An old man said, ‘What moves me most is that you girls from Shandong saved me. Thank you for giving me a second life.’” (Nurse26 Age:35 Female Experience:10).

“The government attached great importance to us and took good care of our families so that we did not have to worry about it.” (Nurse49 Age42 Female Experience:19).

“I felt full of strength because there were my family, friends, and colleagues behind me, as well as my hospital and our powerful motherland!” (Nurse41 Age:29 Male Experience:6).

“I felt warm in my heart when I heard the patient say thanks.” (Nurse23 Age:30 Female Experience:11).

“Thanks to my parents’ understanding, they were very worried and told me over and over again to protect myself. It gave me strength.” (Nurse53 Age:30 Female Experience:5).

“The relationship between nurses and patients is getting much better than before.” (Nurse19 Age:27 Male Experience:5).

#### 3.4.2 Sub-theme 2: hope-the brightest star in the dark

Hope gave people the courage to stick to it. The front-line nurses fighting the pandemic believed victory would come, so they have been working hard.

“The COVID-19 is still going on; however, people are now looking forward to the day when life can resume, and everyone can go outside and breathe freely without a mask. Pandora’s box has been opened, unleashing disaster, illness, and death, but there is one thing still left in it. That is hope. As long as hope is there, all hardships are nothing. Staying together, we will certainly be able to win this epidemic war.” (Nurse14 Age:31 Female Experience:10).

“We were under a lot of physical and mental pressures. However, every time we saw the patient’s eyes, we were full of strength.” (Nurse35 Age:42 Female Experience:23).

“Faced with virus, this is the shared responsibility of medical practitioners.” (Nurse39 Age:40 Female Experience:16).

“After it’s over, I’ll sleep for a week and have a good relaxation. Now I am tired but very happy.” (Nurse34 Age:31 Female Experience:9).

### 3.5 Flow of blessing

Front-line nurses fought hard against the virus, and they gradually entered the front-line state and were able to push themselves to be fully engaged, to maximize their own advantages, and to enter a state of total immersion. They may also become infected, become part of the COVID-19 experience, and gradually understand the feelings of the patients.

#### 3.5.1 Sub-theme 1: medical staff and patients become the whole one

Front-line medical workers worked in isolation wards where the virus was diffused, and the infection rate was very high. Many of them were unfortunately infected by COVID-19. At this time, they were not only medical workers but also witnesses of COVID-19 infection.

“According to the report of China CDC, as of 24: 00 on February 11th, a total of 3,019 medical staff were infected and 1,716 cases were confirmed. Among them, 1,502 medical staff in Hubei Province were diagnosed with infection, accounting for 87.5% of the whole country, got a double experience.” (Nurse3 Age:28 Male Experience:6).

“This moment, we were whole one facing the same enemy, neither medical staffs or patients. That was only one goal.” (Nurse13 Age:43 Female Experience:20).

“Most of the time, roles are separated, whether medical or sick; now it is a double understanding that is both an observer and an experienced person; this moment, COVID-19 makes us stick together.” (Nurse25 Age:37 Female Experience:19).

#### 3.5.2 Sub-theme 2: spiritual happiness

The treatment work of the front-line medical staff was indeed extremely tiresome, and they suffered physical and mental damage. However, they said their psychological pain was tempered by a sense of accomplishment when they saw patients who had escaped the virus and had successfully recovered as a result of their efforts.

“I did not realize it before, and now I really understand Nightingale, founder of nursing just like angel to help others.” (Nurse7 Age:39 Female Experience:18).

“When I saw patients who have been infected with COVID-19 recover, our psychology produced happiness. We have successfully accomplished the task of fighting the epidemic.” (Nurse12 Age:31 Female Experience:11).

“Empathy experience from little childhood joy to great happiness.” (Nurse22 Age:29 Female Experience:7).

#### 3.5.3 Sub-theme 3: empathy experience

The front-line head nurses spent their time in the same space with the patients in the severe epidemic atmosphere. With the increase in working time, they could gradually understand the feelings of the patients, the changes in the patients from their eyes, and even the changes in patients’ emotions.

“I forgot myself in this work, just like same experience whatever they were pleased or sad.” (Nurse1 Age:42 Male Experience:10).

“Most of patients worried about side effects of illness, medical finance burden, family members, and isolation duration at the beginning of the epidemic, however, when they saw those problems was addressed by government or medical staff one by one, such as solutions which were free to cure, most cases became better until recover and went home. I could feel the patient’s emotional changes even I was selflessness.” [*SIC*] (Nurse17 Age:29 Female Experience:9).

“I was sure if everyone have kept in a good pace with whole team, the epidemic will be controlled sooner, so we have need not worried too much.” [*sic*] (Nurse30 Age:39 Female Experience:14).

“Yourself is so little; the big team of cloud medical staff and patient is at the top; your heart beats with patients’ emotion.” (Nurse32 Age:32 Female Experience:9).

## 4 Discussion

This study used role theory as a theoretical framework to explore assigned nurses’ role cognition when supporting Hubei Province in China to fight COVID-19 on the front line. As this was a qualitative phenomenological study based on an empirical sample of 53 participants from Shandong province, our findings may not represent the experiences of those special teams. However, the data provided insight into nurses’ experiences and in-depth views. This also offered the opportunity to explore nurses’ perspectives of role conception.

The first theme reflects what the nurses expected from the aid experience, their role, the difficulty they faced, and how they managed it, as well as the emotional experiences. Research has consistently shown that the expectations of nurses are positive because they believe that assisting Hubei and fighting the epidemic on the front line is their responsibility as nurses, but also as party members, which is an honorable act. As reported (Sohrabi et al., 2019), nurses suffered from a much higher COVID-19 risk than anyone else (Surani et al., 2019). Nightingale believes that the concept of nursing is “the responsibility to protect the health of people and to care for the patient in the best condition.” In the early stages of the outbreak of COVID-19, the nurses in Hubei Province adhered to their work on the front line. Although they also had a certain degree of mental pressure, and some nurses needed to take anti-insomnia drugs and anti-anxiety drugs, they were clear about their responsibilities and interpreted Nightingale’s bravery and dedication with practical actions. They knew that the front line was a dangerous situation and the obligation to turn back to the front line to fight against the epidemic; they overcame all kinds of difficulties because they understood that they were nurses and that a nurse’s duty was to save the dying and heal the wounded.

In terms of role conflicts, studies have shown that the prevalence of depression reported by Chinese healthcare workers during the pandemic was as high as 50%. In the early stage of the COVID-19 outbreak, the mental stress of nurses assisting Hubei Province was more prominent (Dragioti et al., 2022). In the period when the mysterious veil of the virus has not been unveiled, the front-line staff lack the understanding of the virus and the methods to control the spread of the virus, and their hearts are full of fear of the unknown virus. Working conditions on the front lines of the fight against COVID-19 were far from good in the early days of the outbreak. There was a severe shortage of medical staff, each of whom was overworked and often experienced symptoms or negative emotions such as shortness of breath, dizziness, nausea, pain, and burnout. At the same time, due to the lack of protective materials on the front lines of the epidemic, most medical staff did not have enough protective equipment in the hospital. To save the use of protective equipment, they did not eat, drink, or go to the toilet during work hours as much as possible, and most of them wore adult diapers. Such a difficult working environment made them face both physical and psychological stress reactions.

Studies have shown that addressing nurses’ psychological distress is the primary need of nurses (Rathnayake et al., 2021), and carrying out COVID-19 prevention and control education could reduce nurses’ psychological burden and insecurity (Yin and Zeng, 2020). In anticipation of major public health emergencies in the future, it is suggested that the relevant departments of hospitals must improve the medical environment, equip hospitals with enough protective equipment, improve the logistics supply chain, and ensure a sufficient supply of protective medical supplies to better respond to public health emergencies. It is suggested that relevant departments formulate standardized emergency procedures, allocate personnel reasonably, and improve the allocation of human resources and the construction of emergency echelons to prevent emergencies before they occur. At the same time, a flexible dispatch system should be implemented, and various emergency procedures and plans should be actively taught and implemented. We also recommend that front-line workers should be provided with basic living conditions during public health activities, such as a safe environment for medical care and rest, adequate supplies of protective equipment and food, better pay, and honor. Front-line health workers should also be provided with necessary psychological assistance.

As the Spring Festival approached, it was their mission as “soldiers in white clothes” to fight the coronavirus on the front line in Hubei Province during the outbreak of the epidemic instead of spending the important Chinese New Year with their families. They experienced a conflict between their social and family roles, and they worried about their family, and whether their families would have enough daily supplies and protective materials to fight the outbreak during this severe epidemic period. They also worried about how their children and the elderly would manage their lives if they became infected or even died on the front lines. It is suggested that the relevant departments should prioritize logistics and support services to guarantee the wellbeing of the families of the front-line personnel so that the front-line personnel do not have to worry about their families and strive to complete the rescue mission.

Sun emphasized that in the early stages of the crisis, negative emotions dominated, but in the later stages of the crisis, positive emotions gradually emerged (Sun et al., 2020). Similar to this phenomenon, when the front-line nurses gradually adapted to the work of fighting against the epidemic, they gradually adapted to their role as front-line nurses. They received relevant training and knowledge assessment according to the requirements of the organization, mastered certain response measures, and did emergency drills well so they could be relatively more rational and professional in caring for patients infected with COVID-19. They took good care of the infected patients and encouraged them, providing the services of nurses, psychologists, dietitians, cleaners, housekeepers, and so on, doing everything they could to help the patients. They were also good managers, innovators, and educators. They constantly improved the nursing quality. Similar to the research (Seabold et al., 2020), the managers played an important role in the team; they evaluated the situation, promoted safety (Lappalainen et al., 2020), made decisions, supported members, and brought hope and positivity to the whole team.

Comprehensive training, support, and regularly updated guidelines are essential to address the challenges healthcare professionals face in managing the pandemic and to ensure they have the necessary knowledge, skills, and resources (Frenk et al., 2022). The hospital should conduct strict training and evaluation for medical staff to wear and take off protective clothing, learn and evaluate the professional knowledge of infection, master the epidemic mode of infectious diseases and response measures, conduct emergency drills, and be ready to fight at any time to become a reserve force, support the front-line in time in major public health emergencies, and try to solve the problem of shortage of front-line personnel.

In terms of role emotions, many psychological studies have proved that most people are likely to have psychological experiences such as fear, worry, panic, and helplessness when facing a crisis (Zhu, 2020). The COVID-19 pandemic has brought such psychological experiences as fear and anxiety of disease, bereavement, the shock of unemployment, the impact of income, isolation and movement restrictions, the burden of family, confusion and fear of the future, etc. All kinds of mental stress (Chen et al., 2021; Lu et al., 2023) during the COVID-19 pandemic has seriously affected the mental health and wellbeing of the whole society. The nurses in Hubei Province experienced the post-traumatic growth of the sudden infectious disease and felt the correct leadership of the government, the full assistance of the medical staff, the strong support of all walks of life, and the solidarity and mutual assistance of the whole country. They gradually gained the confidence to deal with the disease, united into a fighting group, worked together to overcome the crisis, and won the victory against the COVID-19 epidemic. After growing up with trauma, they have a greater understanding of the feeling of care, a greater learning to take care of others, a better understanding of the truth about the world, a greater understanding of the value and weight of responsibility, a stronger determination to explore the unknown, a greater understanding of the meaning of dedication, and a clearer understanding of their mission.

“Blessing flow” is a concept of happiness put forward by Mi Chail, one of the pioneers of positive psychology, in the 1970s (Peng, 2016). Blessed flow is a feeling, state, and experience, which means that a person shows a strong interest in an activity or thing under the premise of self-awareness and spontaneity and can push himself to be fully invested in it, bringing his own advantages to the maximum, and enter a state of complete immersion (Peng, 2016). Front-line nurses bore the expectations of the whole society and fought hard against the virus at the front line; they got a psychological experience of altruism, flow of blessing, comprehension, understanding and practicing the pain of others and self, and completing the moral internalization of altruism, after that they became more staunch. Support from the team, patients, hospital, and government gave them the motivation to move forward. The relationship between nurses and patients greatly improved. As was reported (Brooks Carthon et al., 2021), the lower the patient satisfaction, the higher the level of nurses’ burnout. Patients’ care and understanding can reduce the level of nurses’ job burnout. Hope is the most important factor for both patients and nurses (Valentine, 2020).

This study significantly contributed to the literature because it demonstrated how non-local nurses sent to Hubei to work perceived their roles as part of a larger narrative of patriotism, duty, solidarity, and hope.

The research object of this study is Chinese nurses in Hubei at the early stage of the outbreak of COVID-19. The research is based on the social background of China. The Chinese government has adopted a national strategy and coordinated all aspects of power to support the prevention and control of the epidemic, which is an important guarantee for society to fight against the COVID-19 epidemic. The unique characteristics of the study population, that is, the front-line nurses fighting against the epidemic in Hubei Province at the early stage of the outbreak, limit the transformability of the findings. The conclusions are not applicable to the role cognition of nurses in ordinary settings. However, the role cognition of the front-line population under crisis events found in this study can provide a reference for the study of the role cognition of the front-line population in major public health emergencies in the future.

## 5 Conclusion

Research has consistently shown that in the face of dangerous epidemics, front-line nurses have high role cognition ability, positive expectations for their role as nurses, good adaptability, and positive role emotions. It was observed that the majority of nurses considered this to be the responsibility of a nurse, as well as that of a party member. Due to the outbreak of the epidemic, front-line hospitals lacked protective equipment and manpower. They were faced with many difficulties, but they were not discouraged. They united as one, helped each other, and tried their best to overcome various difficulties. In addition, their confidence and sense of honor alleviated their role conflict.

More quantitative and qualitative research in the area of COVID-19 patient care can prepare the nursing profession for the new emergent pandemic. Exploring the real experience and role cognition of front-line nurses in response to the epidemic can provide a reference for improving the ability and level of emergency management of public health emergencies and improving the emergency plan for public health emergencies in the future.

## 6 Limitations

This study also has several limitations. First, to mitigate the potential impact of researchers’ preconceived notions on the results, researchers have taken precautionary measures before the start of the study to minimize the impact on the results by setting aside prior knowledge and bias and addressing the problem of reflexivity. Second, the data were collected from nurses’ self-reports of their experiences and feelings, and the findings may be modified by cultural differences and different sociodemographic characteristics. Third, in the early stage of the outbreak, due to the restriction of prevention and control measures and the limitation of research sites, the sample data of this study finally came from six public hospitals in Shandong Province. However, China’s geographical and social environment is relatively complex, and hospitals of different sizes and levels may have certain differences in the results. Therefore, the applicability and generalization of the conclusions of this study need to be further verified. Finally, this study is affected by social expectation bias and personal value judgment, so respondents will hide their true thoughts to a certain extent, which will have a certain impact on our research results. In summary, a larger scale multi-center qualitative and quantitative study in the field of COVID-19 patient care is needed in the future to expand the confidence of the existing analytical results of this study.

## Data availability statement

The raw data supporting the conclusions of this article will be made available by the authors, without undue reservation.

## Ethics statement

The studies involving humans were approved by Science Research of Qilu Hospital of Shandong University, approved document number KYLL-2020-258. The studies were conducted in accordance with the local legislation and institutional requirements. The participants provided their written informed consent to participate in this study.

## Author contributions

XZ: Data curation, Writing – original draft. YW: Writing – original draft, Data curation. YC: Writing – review & editing. HY: Investigation, Methodology, Writing – review & editing. XL: Writing – review & editing.
