# Supplementary material for: Role cognition of assigned nurses supporting Hubei Province in the fight against COVID-19 in China: a hermeneutic phenomenological study
Source: Front Psychol. 2024 Feb 29;15:1287944. doi: 10.3389/fpsyg.2024.1287944 (PMC10939063; doi:10.3389/fpsyg.2024.1287944)
Supplement: Supplementary file 1 [file Data_Sheet_1.docx]

**Table of contents of supplementary material**

***[Supplementary Table 1. Consolidated criteria for reporting qualitative studies (COREQ): 32-item checklist.](#_Toc144720576)***

[2](#_Toc144720576)

[Supplementary Table 2. The testing and revision process of the interview guide. 7](#_Toc144720577)

***[Supplementary Table 3. Themes, sub-themes and coding examples.](#_Toc144720578)***

[8](#_Toc144720578)

**Supplementary Table 1. Consolidated criteria for reporting qualitative studies (COREQ): 32-item checklist**

| **No** | **Item** | **Guide questions/description** | **Response** |
| --- | --- | --- | --- |
| **Domain 1: Research team and reflexivity** | | | |
| Personal Characteristics | | | |
| 1 | Interviewer/facilitator | Which author/s conducted the interview or focus group? | Hailing Yang , Yuanyuan Chen, Yaqian Wang and Xu Zhang |
| 2 | Credentials | What were the researcher's credentials? E.g. PhD, MD | Master of Public Health and master of nursing specialist |
| 3 | Occupation | What was their occupation at the time of the study? | Nurse management and master of nursing specialist |
| 4 | Gender | Was the researcher male or female? | All researchers are female. |
| 5. | Experience and training | What experience or training did the researcher have? | The correspondence author have worked in nursing for 17 years,include nursing management for 8 years in nursing department.  The other corresponding author is studying for a PhD, and the other two are studying for a masters.  All of them have trained about qualitative research. |
| Relationship with participants | | | |
| 6 | Relationship established | Was a relationship established prior to study commencement? | Yes. A relationship was established when the researcher contacted the participants. |
| 7 | Participant knowledge of the interviewer | What did the participants know about the researcher? e*.g. personal goals, reasons for doing the research* | After introducing the purpose of the study, participants understood that their participation was to help explore the status of role perception among front-line nurses during the outbreak, and they also enjoyed sharing their feelings with our managers. |
| 8 | Interviewer characteristics | What characteristics were reported about the interviewer/facilitator? e.g. Bias, assumptions, reasons and interests in the research topic | The interviewer, based on previously  established relationship with participants, provided a conducive and trusting environment for participants to share their experiences.  Bias maybe from single-center research，it need more survey to improve the quality. |
| **Domain 2: study design** | | | |
| Theoretical framework | | | |
| 9 | Methodological orientation and Theory | What methodological orientation was stated to underpin the study? *e.g. grounded theory, discourse analysis, ethnography, phenomenology, content analysis* | Phenomenology  *This study used the phenomenological hermeneutics method to deeply explore the real coping experience and role recognition process of front-line nurses fighting against COVID-19 in Hubei Province. Phenomenological hermeneutics was chosen because this can be used to test the basic meaning of experience-based phenomena when interpreting data.* |
| Participant selection | | | |
| 10 | Sampling | How were participants selected? *e.g. purposive, convenience, consecutive, snowball* | Purposive  *By purposive sampling method and maximum difference sampling strategy front-line nurses from 6 public hospitals in Shandong Province were selected.* |
| 11 | Method of approach | How were participants approached? e*.g. face-to-face, telephone, mail, email* | Face-to-face and Telephone  *Face-to-face interviews were conducted in a meeting room with good privacy to ensure a quiet, spacious and undisturbed environment, with only interviewers and interviewees present. The telephone interview was conducted in a quiet room with good Internet communication signal.* |
| 12 | Sample size | How many participants were in the study? | 53 Participants  *After semi-structured interviews with 51 participants, data saturation, a state in which no new information is available and coding becomes infeasible, was reached, after which two additional participants were interviewed.* |
| 13 | Non-participation | How many people refused to participate or dropped out? Reasons? | 0 participants  *All 53 patients agreed to participate in the study.* |
| Setting | | | |
| 14 | Setting of data collection | Where was the data collected? e*.g. home, clinic, workplace* | Workplace  *Face-to-face interviews were conducted in a meeting room with good privacy to ensure a quiet, spacious and undisturbed environment, with only interviewers and interviewees present. The telephone interview was conducted in a quiet room with good Internet communication signal.* |
| 15 | Presence of non-participants | Was anyone else present besides the participants and researchers? | No  *Face-to-face interviews were conducted in a meeting room with good privacy to ensure a quiet, spacious and undisturbed environment, with only interviewers and interviewees present. The telephone interview was conducted in a quiet room with good Internet communication signal.* |
| 16 | Description of sample | What are the important characteristics of the sample? *e.g. demographic data, date* | Demographic data  *The Demographic characteristics of the sample are reported in Table 1.* |
| Data collection | | | |
| 17 | Interview guide | Were questions, prompts, guides provided by the authors? Was it pilot tested? | Based on document analysis,questions, prompts, guides were provided by the authors,before it have pilot tested with 2 participants.  *The interview outline was finalized based on previous literature review, theoretical analysis, consultation with experts in related fields, and discussion and revision based on pre-interview results.* |
| 18 | Repeat interviews | Were repeat interviews carried out? If yes, how many? | No |
| 19 | Audio/visual recording | Did the research use audio or visual recording to collect the data? | Yes  *During the interview, recording equipment was used to record the interview process.* |
| 20 | Field notes | Were field notes made during and/or after the interview or focus group? | Yes  *Field notes were made during the interview, after the interview, the records were sorted out and checked.* |
| 21 | Duration | What was the duration of the interviews or focus group? | *Interviews were recorded and varied in duration, with an average duration of approximately 35 minutes. Interviews lasted 30 to 40 minutes.* |
| 22 | Data saturation | Was data saturation discussed? | Yes  *The sample size was set to interview two more respondents after their data appeared repeatedly, and no new themes were presented as standards during data analysis.* |
| 23 | Transcripts returned | Were transcripts returned to participants for comment and/or correction? | Yes  *Finally, the resulting theme structure is returned to the participants for examination, and if there is a bias, the researcher must step back to the analysis from the first step.* |
| **Domain 3: analysis and findings** | | | |
| Data analysis | | | |
| 24 | Number of data coders | How many data coders coded the data? | Four  *XZ,YQW,HLY, and YYC jointly participated in data analysis and coding.* |
| 25 | Description of the coding tree | Did authors provide a description of the coding tree? | Yes  *Provided in the supplementary table 3.* |
| 26 | Derivation of themes | Were themes identified in advance or derived from the data? | Derived from the data. |
| 27 | Software | What software, if applicable, was used to manage the data? | NVivo 12.0  *The study used NVivo 12.0 software for importing, organizing, and exploring data for analysis. NVivo is a Computer-Assisted Qualitative Data Analysis Software.* |
| 28 | Participant checking | Did participants provide feedback on the findings? | Yes  *In the analysis of the data after the interviews, the description of the participants was considered and it was ensured that the opinions of all participants were reflected in the results section.* |
| Reporting | | | |
| 29 | Quotations presented | Were participant quotations presented to illustrate the themes / findings? Was each quotation identified? e*.g. participant number* | Yes, quotations are included in the results section.  Each quotation was indexed using participant number, gender, age and experience in the case of patients. |
| 30 | Data and findings consistent | Was there consistency between the data presented and the findings? | Yes |
| 31 | Clarity of major themes | Were major themes clearly presented in the findings? | Yes  *See Results and table 2.* |
| 32 | Clarity of minor themes | Is there a description of diverse cases or discussion of minor themes? | Yes  *See Results and table 2.* |

**Supplementary Table 2. The testing and revision process of the interview guide**

| **No** | **First draft of interview outline** | **Revision suggestions**  **(expert meeting + pre-interview)** | **Final draft of interview outline** |
| --- | --- | --- | --- |
| 1 | As assigned nurses, why did you choose to support Hubei province fighting COVID-19? | To establish a closer connection with the interviewees, it is recommended to avoid overly formal and written expressions when formulating questions. | Why did you choose to go to Hubei as a backup? |
| 2 | Did you have any good expectations for going to Hubei? | Remove the interview outline with suggestive and directive descriptions. | What were your expectations of going to Hubei? |
| 3 | What kind of person do you see yourself as? | No modification | What kind of person do you see yourself as? |
| 4 | What difference did it make for you and your family? | No modification | What difference did it make for you and your family? |
| 5 | What was your motivation to work? | No modification | What was your motivation to work? |
| 6 | What preparations did you and your family members make for going to Hubei? | Focus on the experience and feelings of front-line nurses, and reduce questions about their family members. | What preparations did you make to go to Hubei as support staff? |
| 7 | What difficulties did you encounter at work? | Dig deep into the entire experience and process of how the interviewees overcome difficulties and solve problems. | What difficulties did you encounter at work and how did you overcome them? |
| 8 | What changes have you made? | No modification | What changes have you made? |
| 9 | How was your relationship with your colleagues and patients at work? | No modification | How was your relationship with your colleagues and patients at work? |
| 10 | What was the biggest change you’ve faced? | The meaning of the problem description was unclear, and the patient's understanding was biased. | What was the biggest change you’ve faced since you left your hometown? |
| 11 | Please describe one thing that you think was most meaningful. | No modification | Please describe one thing that you think was most meaningful. |
| 12 | What things were the most impressive for you? | No modification | What things were the most impressive for you? |
| 13 | How did you feel? | No modification | How did you feel? |
| 14 | What do you wish for when the epidemic is over? | No modification | What do you wish for when the epidemic is over? |

**Supplementary Table 3.** **Themes, sub-themes and coding examples**

| **Themes** | **Sub-themes** | **Coding examples** |
| --- | --- | --- |
| **Role Expectations** | **I want to be the soldier in White** | Heroes in harm's way, duties of nurses, the mission of fighting against the epidemic, becoming a hero, winning the battle against the epidemic |
|  | **It is my mission as a party member** | Common beliefs, needs of the organization, the vanguard and exemplary role of party members, responding to the call of the Party Central Committee, shouldering the mission of the Party and the country |
|  | **It’s my honor to aid front-line nursing** | Conveying the spirit of Nightingale, the glory of a lifetime, remembering the medical oath, fighting for people's health, defending the country with our lives, active registration, carrying the trust and entrust of the people |
|  | **We are family** | Integration of family and country, giving up the little family for everyone, Chinese national spirit, the responsibility of Chinese People, national cohesion and fighting capacity, national unity of the people |
| **Role Conflicts** | **It’s not easy to work in protective gear** | Blurred vision, light-headedness, nausea and chest tightness, dyspnea, sensory limitation, drenched in sweat, closed and stuffy, pressure damage to the skin, pain under pressure, delayed action |
|  | **I’m sorry for my family I neglected** | Funerals for absent relatives, postponing lover's wedding, lack of companionship and care for loved ones, lack of parenting and attachment to children |
|  | **Stretched thin** | Overwork, high risk of infection, dignified working atmosphere, urgent rescue work, difficult working conditions, disease uncertainty, staff shortage, inner anxiety, heavy work pressure |
| **Role Adaptation** | **Overcome the inadaptability of protective gears** | Adjusting breathing rhythm, relieving discomfort symptoms, adapting to work rhythm, practicing the use of protective gear, adjusting mindset, mastering operational skills |
|  | **A team leader is like the sun in the ICU** | Responsibilities of the manager, coordinating team work, dealing with emergencies, assessing the situation, making team decisions, conveying beliefs and hopes, ensuring basic materials, taking care of and caring for team members, ensuring the safety of team members |
|  | **More than just a nurse** | Psychological counselor, health care assistant, nutritionist, social work workers, hospital infection control personnel, cleaning staff, inventor, property manager, sign language interpreters, friends, health educator, nurse researcher |
| **Role Emotions** | **You never walk alone** | Full support from society, solid guarantee from the country, strong support from hospitals, understanding and encouragement from family members, cooperation, recognition from patients, companion and encouragement from colleagues |
|  | **Hope-The brightest star in the dark** | Believing in the arrival of victory, looking forward to reunion and happiness, imagining a Better Future, the power of hope, the hope of living, the spirit of never giving up |
| **Flow of Blessing** | **Medical staff and patients become the whole one** | Dual Role Experience, alignment of goals, common aspirations, working together |
|  | **Spiritual happiness** | A sense of achievement in completing tasks, the happiness of the patient's improved condition, the sense of honor of saving lives and helping the injured, the warmth and affection of human love, a solid sense of government and social support |
|  | **Empathy experience** | Empathizing with others, placing oneself in others' position, going through thick and thin together, making concerted efforts, overcoming difficulties together, empowering patients |
